# Artifact-Aware Fungal Detection in Dermatophytosis: A Transformer-Based Approach for KOH Microscopy

**DOI:** 10.3390/bioengineering13050591

**Published:** 2026-05-21

**Authors:** Rana Gursoy, Abdurrahim Yilmaz, Baris Kizilyaprak, Esmahan Caglar, Burak Temelkuran, Huseyin Uvet, Ayse Esra Koku Aksu, Gulsum Gencoglan

**Affiliations:** 1 Department of Mechatronics Engineering, Yildiz Technical University, Istanbul 34220, Turkey; rana.gursoy@yildiz.edu.tr; 2 Division of Systems Medicine, Department of Metabolism, Digestion and Reproduction, Imperial College London, London SW7 2AZ, UK; burak.temelkuran@imperial.ac.uk; 3 Department of Dermatology and Venereology, Istanbul Research and Training Hospital, Istanbul 34098, Turkey; brskzlyprk@icloud.com (B.K.); ayseesra.kokuaksu@sbu.edu.tr (A.E.K.A.); 4 Department of Bioengineering, Yildiz Technical University, Istanbul 34220, Turkey; esmaacaglar64@gmail.com; 5 Artificial Intelligence Research and Application Center (YZAUM), Istinye University, Istinye 34396, Turkey; 6 Department of Dermatology and Venereology, Medicana Atakoy Hospital, Istanbul 34158, Turkey; ggencoglan@gmail.com

**Keywords:** deep learning, dermatophytosis, koh microscopy, object detection

## Abstract

Dermatophytosis is commonly assessed using potassium hydroxide (KOH) microscopy, yet accurate recognition of fungal hyphae is hindered by preparation-related artifacts, heterogeneous keratin clearance, and notable inter-observer variability. This study presents a transformer-based object detection framework using the RT-DETR architecture for precise, query-driven localisation of fungal structures in high-resolution KOH images. A dataset of 2540 routinely acquired microscopy images was manually annotated using a multi-class strategy that explicitly distinguishes fungal elements from confounding artifacts, enabling the model to actively suppress false detections arising from visually similar mimics. To assess architectural trade-offs, RT-DETR was benchmarked against two CNN-based detectors (YOLOv11 and Faster R-CNN) under identical training and inference conditions. Five-fold stratified cross-validation was performed, and each fold-level model was evaluated on the same independent held-out test set (*n* = 254). Across the five evaluations, RT-DETR achieved a mean AP@0.50 of 89.73%±1.48%, a mean recall of 0.831±0.011, and a mean precision of 0.921±0.014. At the image level, the model achieved a mean sensitivity of 0.989±0.022 on the independent test set, with a mean of 0.2±0.4 missed positive cases across the five evaluations. These results demonstrate the technical feasibility of a transformer-based artificial intelligence (AI) system as a decision-support aid for fungal region detection in KOH microscopy, pending prospective multi-center validation to establish clinical generalisability.

## 1. Introduction

Dermatophytosis is a common fungal infection of the nails and body. Early and accurate diagnosis is of critical clinical importance, as it directly influences treatment success [1]. Conventional diagnostic methods primarily include direct microscopic examination with KOH and fungal culture, both of which remain widely used in clinical practice. However, these approaches are limited by prolonged analysis times, relatively low sensitivity, and significant inter-observer variability, particularly in microscopic evaluation [2,3].

The KOH preparation, one of the most frequently employed methods for microscopic confirmation, dissolves keratin and enhances the visibility of fungal hyphae and spores. However, direct KOH microscopy often presents diagnostic challenges due to residual keratin, air bubbles, debris, and artifactual structures that may resemble fungal elements, contributing to inter-observer variability and reduced sensitivity [4,5]. Building a robust detection system requires addressing these inherent variabilities in samples prepared with KOH. In particular, artifacts that visually mimic fungal structures represent a major source of diagnostic ambiguity, not only for human observers but also for automated systems trained without explicit artifact-aware strategies. These artifacts also complicate automated analysis, particularly for object-detection models that rely on accurate spatial localisation [6,7].

In recent years, AI and deep learning methodologies have increasingly been adopted to address diagnostic challenges in medical image analysis, particularly in settings where subtle, low-contrast structures must be identified within complex visual backgrounds [8,9]. Through their ability to learn complex representations from large datasets, these models can detect subtle and low-contrast patterns that are not readily discernible under conventional visual inspection [10,11]. Deep learning has achieved expert-level performance across multiple medical imaging domains, including radiology and digital pathology [12]. In dermatology specifically, state-of-the-art models have demonstrated diagnostic accuracy comparable to, and in some cases exceeding, that of experienced dermatologists in the recognition of skin lesions [13,14,15].

To place fungal hyphae detection in the broader context of microscopic image analysis, recent deep learning applications in related domains are briefly reviewed below. Microscopic image analysis has likewise become an important focus within medical AI, where deep learning models have been successfully applied to detect small, morphologically variable, and low-contrast biological structures [16,17]. Strong performance has also been demonstrated in blood-smear microscopy, including malaria parasite detection using convolutional neural network (CNN)-based or hybrid architectures [18]. Convolutional and transformer-based models have further enabled reliable identification of parasitic forms and accurate detection of abnormal epithelial cells in cervical cytology [7,19]. In addition, hybrid deep feature fusion strategies, such as those implemented in the DeepCervix framework, have advanced automated cervical cell analysis [20]. Comparable progress in histopathology, including precise nuclei segmentation and classification, highlights the capacity of modern object-detection frameworks to localise fine, irregular, and artifact-obscured targets within complex visual fields [6,21]. These shared imaging characteristics (small object size, low contrast, complex textures, and the presence of distracting artifacts) closely mirror the challenges observed in KOH microscopy, making fungal hyphae a suitable and clinically relevant target for object-detection approaches.

Deep learning-based studies on dermatophytosis have proliferated rapidly in recent years. Yılmaz et al. (2022) used CNN-based models on KOH microscopy images and achieved 96.2% accuracy in classifying infected versus healthy samples [22]. Jansen et al. (2022) employed a U-Net-based segmentation model on histologic nail sections, identifying fungal structures with a Dice score of 91% [23]. Decroos et al. (2021) developed a deep learning model for histopathologic sections and obtained 92.5% sensitivity, proving non-inferiority to conventional histopathologic diagnosis [24]. Koo et al. applied a YOLOv4-based object detection model to KOH microscopy images of superficial fungal infections, demonstrating that automated hyphae detection with bounding boxes is feasible in routine practice [25]. However, this study was limited to superficial mycoses rather than KOH preparations, relied on an earlier CNN architecture, and did not address the substantial artifactual heterogeneity characteristic of dermatophytosis microscopy. Beyond single-architecture studies, recent research in biomedical object detection has emphasized the importance of comparative evaluation across different detection paradigms. Modern single-stage convolutional detectors such as YOLOv11 [26] and classical two-stage region-proposal networks such as Faster R-CNN [27] represent the principal CNN-based baselines against which transformer-based frameworks are benchmarked, yet their relative performance in artifact-rich microscopy settings has not been systematically examined. Consequently, despite increasing interest in automated analysis, most available research still focuses on image-level classification or pixel-wise segmentation, and object-level detection approaches in routine KOH microscopy have not yet been systematically investigated. Crucially, although previous studies have addressed fungal detection using deep learning, relatively limited attention has been given to explicitly modeling artifactual structures as a distinct detection class in KOH microscopy datasets. Given the well-recognized diagnostic challenges posed by visually similar mimickers, incorporating artifacts as a separate class may improve the robustness of automated detection systems in routine microscopy settings [28].

From a clinical perspective, spatial localisation of fungal elements may offer additional information for estimating fungal burden and guiding treatment decisions, highlighting the insufficiency of image-level classification. While visually similar artifacts in KOH preparations frequently confound conventional local feature-based detectors, transformer-based architectures leverage global self-attention to effectively discriminate between biological structures and mimics, as evidenced by recent success in similar microscopic tasks [29,30,31].

In this study, we developed and evaluated an AI-based detection system for the automated and precise localisation of fungal elements in routine KOH microscopy. All microscopic images were obtained using this routine preparation technique, thus capturing the heterogeneity, noise, and artifact patterns commonly encountered in real-world clinical practice, which are known to challenge the generalizability of AI-based systems [8,10,32]. Based on these considerations, we selected the RT-DETR (Real-Time Detection Transformer) model [33] as the object detection backbone. This study involved constructing an expert-verified and carefully curated dataset, where both fungal elements indicative of dermatophytosis and confounding artifactual structures were explicitly annotated as distinct classes. By training the RT-DETR model to simultaneously detect and distinguish these entities under standardized conditions, we assessed its performance using established detection metrics. Through this framework, we aimed to provide a fully automated and reproducible approach for the localisation of fungal elements in routine KOH microscopy that remains robust against real-world artifactual interference.

## 2. Materials and Methods

**Figure 1:**
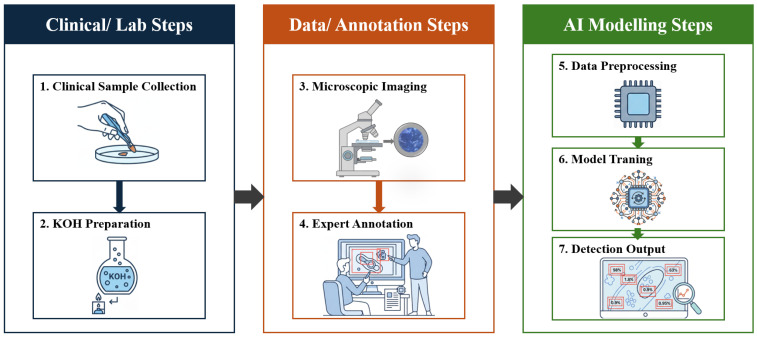
Schematic overview of the study workflow: clinical sample collection, KOH preparation, microscopic imaging, expert annotation, and artificial intelligence modelling steps.

The complete study pipeline from clinical specimen collection and KOH preparation, through high-resolution microscopic imaging and expert bounding-box annotation, to object detection model training, validation, and inference is schematically illustrated in Figure 1.

### 2.1. Clinical Sample Collection and Microscopic Imaging

The study involving human-derived specimens was approved by the Ethics Committee for Clinical Research of Istanbul Training and Research Hospital under protocol number 2011-KAEK-50, with approval number 20. Human scrapings were collected at Istanbul Research and Training Hospital under sterile conditions from patients with clinically and/or mycologically confirmed dermatophytosis. The specimens were incubated in 10–20% KOH solution to dissolve keratin, thereby enhancing the visibility of fungal hyphae and spores; nail specimens were incubated for 12 hours, whereas skin specimens were incubated for 1 hour. The cleared preparations were subsequently examined and imaged at high resolution using light microscopes.

A total of 2540 microscopic images (2048×2048 pixels) were acquired. These images were obtained from 100 clinical specimens collected from 60 patients diagnosed with dermatophytosis. Multiple microscopic fields were captured from each specimen during routine microscopic examination, resulting in the total set of 2540 high-resolution microscopy images used for model development and evaluation. All images were reviewed and subjected to an initial quality-control assessment by two clinicians (Gulsum Gencoglan (G.G.) and Baris Kizilyaprak (B.K.)), who subsequently performed the manual annotation. All annotations were reviewed by both clinicians experienced in dermatological microscopy. In cases of disagreement, the images were jointly re-evaluated and a consensus decision was reached. The descriptive statistics and class distribution of the resulting dataset are summarized in Table 1.

**Figure 2:**
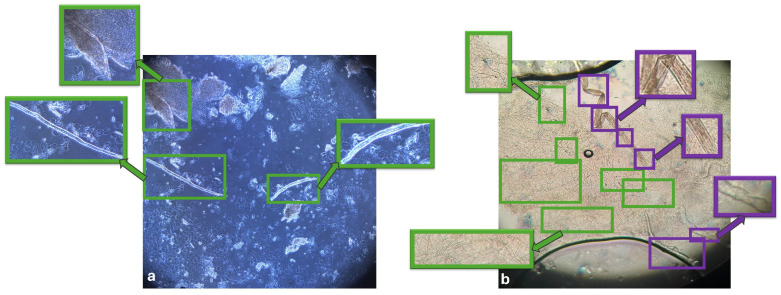
(**a**,**b**) Representative KOH microscopy image illustrating the multi-class annotation strategy. Fungal hyphae are marked with green bounding boxes and and confounding artifacts are marked with purple boxes.

The annotation process adopted a multi-class strategy to address visual ambiguity in KOH microscopy. In addition to fungal elements, clinically relevant artifactual structures (such as keratin debris and fibres) were annotated as a separate class to facilitate model discrimination between true fungal structures and visually similar mimics. By deliberately labeling these mimics rather than treating them as background, the we designed to teach the model to actively discriminate between true fungal structures and the diagnostic challenges frequently encountered in routine KOH microscopy. A representative example of these multi-class expert annotations is shown in Figure 2.

### 2.2. Detection Architecture Overview and Experimental Design

Object detection in KOH microscopy presents a distinctive set of visual challenges: fungal hyphae are thin, low-contrast, and morphologically variable, while preparation-related artifacts, including keratin debris, synthetic fibres, refractive edges, and air bubbles, frequently resemble genuine fungal structures at the spatial scales relevant to bounding-box detection. To assess whether the design paradigm of the detection architecture meaningfully influences performance under these conditions, three distinct model families were evaluated: a transformer-based end-to-end detector (RT-DETR), a contemporary single-stage convolutional detector (YOLOv11), and a classical two-stage region-proposal network (Faster R-CNN).

RT-DETR [33] was selected as the primary model based on its hybrid CNN–Transformer encoder–decoder architecture, which integrates multi-scale convolutional feature extraction with global self-attention across the full image field. This design enables the model to contextualise each candidate detection within the surrounding tissue environment, a property hypothesised to be advantageous when discriminating structurally similar fungal and non-fungal structures. The attention-driven, query-based detection head performs direct bounding-box regression and classification without non-maximum suppression, removing a source of post-processing sensitivity that can affect recall in densely annotated microscopic fields. Training procedures, hyperparameter configuration, and augmentation strategies for RT-DETR are described in detail in below.

YOLOv11 was included as a contemporary convolutional baseline, representing a modern single-stage detection paradigm widely used in real-time computer vision applications [26]. Its architecture combines efficient convolutional feature extraction with an enhanced feature-pyramid neck, enabling rapid inference while maintaining competitive localisation accuracy. Including YOLOv11 allows assessment of whether transformer-based global attention mechanisms provide measurable advantages over modern convolutional feature hierarchies in the context of artifact-rich KOH microscopy.

Faster R-CNN was included as a classical two-stage region-proposal detector that historically formed the foundation of many medical object-detection systems [27]. Its region-proposal network first generates candidate object regions, which are subsequently refined and classified by a second-stage detection head. This architecture has been widely applied to tasks involving spatially sparse and morphologically heterogeneous objects. Evaluating Faster R-CNN therefore provides a reference baseline representing earlier convolutional detection frameworks.

### 2.3. Artificial Intelligence Model and Training Procedure

**Figure 3:**
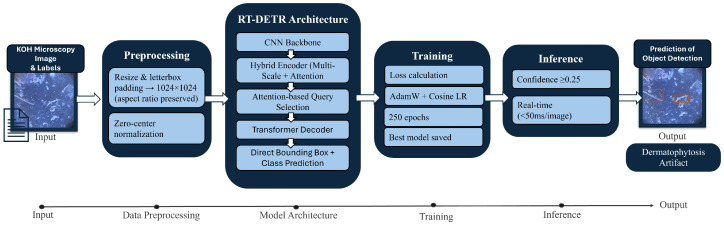
Overview of the fungal detection pipeline. KOH microscopy images are preprocessed and passed through the RT-DETR architecture, which combines a CNN backbone, hybrid encoder, attention-based query selection, and a transformer decoder to generate bounding box predictions for fungal elements and confounding artifacts.

All three architectures (RT-DETR, YOLOv11, and Faster R-CNN) were trained on identical dataset partitions using the same two-class annotation scheme (fungal elements and confounding artifacts). The following describes the architectural configuration, training procedure, and inference settings specific to RT-DETR as the primary model. Fungal element detection in KOH microscopy images was performed using the RT-DETR model, an end-to-end transformer-based object detector. The model employs an attention-driven query selection strategy within a hybrid CNN-Transformer encoder-decoder pipeline, enabling direct bounding box regression and classification. Combined with multi-scale feature fusion and efficient query refinement, the proposed architecture facilitates the detection of small, thin, and irregularly shaped fungal structures, such as hyphae and arthroconidia, while simultaneously distinguishing them from confounding artifactual mimics in dermatological microscopy. The overall model workflow, including preprocessing, architectural components, and inference logic, is illustrated in Figure 3.

All experiments were conducted using the Ultralytics implementation of the RT-DETR model (Ultralytics implementation, version 8.2.70), with the RT-DETR-L configuration pre-trained on the COCO dataset serving as the initialisation. To ensure reproducibility, we note that training was performed on a workstation equipped with an NVIDIA GeForce RTX 4090 24 GB (NVIDIA Corporation, Santa Clara, CA, USA). To obtain statistically robust performance estimates and address the sensitivity of single-split evaluation in moderate-sized clinical imaging datasets, all three architectures were trained under a five-fold stratified cross-validation protocol applied to the training and validation pool (*n* = 2286 images). Stratification was performed at the image level to preserve the proportion of annotated positive and negative images across all folds. In each iteration, four folds (≈1829 images) were used for training and one fold (≈457 images) for validation. Model checkpoints were selected based on AP@0.50:0.95 on the fold-level validation partition. The independent held-out test set (*n* = 254 images) was excluded from all training and validation procedures and was used exclusively for final performance evaluation. All reported metrics represent the mean and standard deviation of the five fold-level models evaluated on this common independent test set.

Model training followed the hyperparameters and optimization strategy defined in the custom training pipeline. Specifically, the model was trained for up to 250 epochs using the AdamW optimizer, with an initial learning rate of $5\times 10^{-4}$, cosine-annealed decay with warm-up, a batch size of 8, and mixed-precision training to reduce GPU memory consumption. Loss functions were weighted following standard practice (box = 7.5, classification = 1.0), and early stopping with a patience of 50 epochs was applied to prevent overfitting.

Given the morphological sensitivity of fungal hyphae and the need to preserve distinct artifact features, aggressive augmentations such as MixUp were intentionally disabled. Standard geometric variations were utilized, including horizontal flipping (p=0.2), scaling (±20%), translation (±5%), and minor rotations (±2°). These choices ensured preservation of clinically relevant fungal morphology while still providing sufficient variability for robust learning.

Model selection was based on validation performance measured by AP@0.50:0.95, which reflects detection consistency across different intersection-over-union thresholds for all classes. During inference, predictions were filtered using a confidence threshold of 0.25. Due to its transformer-based matching mechanism, the model enables real-time prediction (<50 ms per image on the NVIDIA RTX 4090 GPU) and improves reliability when fungal structures appear in dense or overlapping formations. This end-to-end inference pipeline is summarized in Figure 3, which visualizes the progression from microscopy image preprocessing to final multi-class fungal region localisation.

### 2.4. Baseline Model Evaluation

All three architectures were trained under the same five-fold stratified cross-validation protocol on the training and validation pool (*n* = 2286 images) using the identical two-class annotation scheme (fungal elements and artifacts). To facilitate direct architectural comparison, key training parameters were harmonized across the three frameworks where applicable. Specifically, all models were trained for up to 250 epochs with an input resolution of 1024 × 1024 pixels, a batch size of 8, and COCO pretrained weights as initialization. The initial learning rate was set to 5 × 10^−4^ with cosine annealing and early stopping (patience = 50 epochs). Architecture specific components without direct equivalents across frameworks (e.g., the transformer query mechanism in RT-DETR, the distribution focal loss in YOLOv11, or the region proposal network in Faster R-CNN) were kept at their recommended default settings. Each fold-level model was then evaluated on the same independent held-out test set (*n* = 254). To ensure a fair comparison, the same confidence threshold (0.25) was applied to all architectures, and detection metrics were computed using identical intersection-over-union criteria (AP@0.50 and AP@0.50:0.95). Inference speed was benchmarked under identical hardware conditions on the NVIDIA RTX 4090 GPU. This consistent evaluation protocol ensures that observed performance differences reflect architectural characteristics rather than differences in experimental conditions.

All reported metrics represent the mean and standard deviation of the five fold-level models evaluated on this common independent test set. This design was adopted to quantify training stability rather than to perform ensemble inference. Each of the five independently trained models was evaluated on the same held-out test set solely to estimate the variance in performance attributable to training randomness and fold-specific data composition. The held-out test set was not used at any stage of model selection or hyperparameter tuning and was accessed only once per fold-level model for final performance reporting.

## 3. Results

Table 2 summarises the object-level detection and image-level classification performance of all three architectures, reported as the mean and standard deviation across five models trained on different cross-validation folds and evaluated on the same independent held-out test set.

At the object level, RT-DETR achieved a mean precision of 0.921±0.014, a mean recall of 0.831±0.011, a mean F1-score of 0.874±0.012, a mean AP@0.50 of 89.73±1.48%, a mean AP@0.50:0.95 of 76.85±1.22%, and a mean IoU of 0.838±0.009 on the independent test set. YOLOv11 achieved a mean precision of 0.812±0.016, a mean recall of 0.842±0.012, a mean F1-score of 0.827±0.013, a mean AP@0.50 of 81.34±1.52%, a mean AP@0.50:0.95 of 61.28±1.41%, and a mean IoU of 0.846±0.008. Faster R-CNN achieved a mean precision of 0.333±0.013, a mean recall of 0.800±0.008, a mean F1-score of 0.470±0.015, a mean AP@0.50 of 51.54±1.23%, a mean AP@0.50:0.95 of 27.47±1.01%, and a mean IoU of 0.750±0.008.

Image-level classification performance was assessed by aggregating object-level detections per image. A case was classified as positive if at least one fungal element was detected above the confidence threshold (0.25). On the independent held-out test set (n=254), RT-DETR achieved a mean sensitivity of 0.989±0.022, a mean specificity of 0.970±0.005, a mean accuracy of 0.976±0.008, a mean F1-score of 0.967±0.012, and a mean of 0.2±0.4 missed positive cases across the five evaluations. YOLOv11 achieved a mean sensitivity of 0.767±0.014, a mean specificity of 0.951±0.008, a mean accuracy of 0.886±0.012, a mean F1-score of 0.826±0.013, and a mean of 4.2±1.1 positive cases. Faster R-CNN achieved a mean sensitivity of 0.522±0.021, a mean specificity of 0.896±0.013, a mean accuracy of 0.765±0.018, a mean F1-score of 0.610±0.019, and a mean of 8.6±1.5 positive cases.

## 4. Discussion

This study presents an object detection model for localising fungal structures in KOH microscopy images of dermatophytosis. Previous deep learning work in this field has largely focused on image-level classification of infected versus non-infected samples or pixel-wise segmentation of fungal elements [22,23,24]. In contrast, our approach targets explicit object-level localisation with bounding boxes, utilizing a multi-class detection strategy to distinguish true fungal elements from confounding artifacts, thereby aiming to provide interpretable and explainable visual cues on routine microscopy images.

Modern detection architectures have recently been applied to the automated analysis of fungal infections. Koo et al. employed a YOLOv4-based one-stage convolutional detector for hyphae detection in KOH microscopy images, demonstrating that automated bounding-box localisation is feasible in clinical practice [25]. While their work provided an important proof of concept for CNN-based object detection, YOLOv4 relies on convolutional feature hierarchies and predefined anchor mechanisms, which represent a different design paradigm compared to transformer-based detectors. Moreover, their study focused on superficial mycoses under relatively controlled imaging conditions, with less visual complexity than is typically encountered in KOH preparations. In contrast, the present study evaluates the RT-DETR model, a transformer-based detector that performs end-to-end object detection, using attention-driven query selection and global contextual reasoning. This architecture was evaluated in high-resolution KOH microscopy settings, where fungal hyphae appear small, low-contrast, and are often embedded within dense backgrounds containing keratin debris and preparation-related artifacts. By explicitly modelling mimics, such as fibres and air bubbles, a distinct class during training, our model is designed to incorporate global contextual reasoning, which may help reduce false positive detections in artifact-rich KOH microscopy settings. From a computational perspective, the model enables stable real-time inference in our experimental setting (below 50 ms per image), while maintaining a simplified detection pipeline without post-processing. These characteristics support not only efficient image-level analysis but also scalability to large microscopic fields, which may be advantageous for time-sensitive microscopic analysis in experimental settings. Taken together, while YOLOv4-based approaches have demonstrated the feasibility of automated hyphae detection, alternative detection frameworks that emphasize global contextual information and end-to-end prediction may offer complementary advantages in artifact-rich KOH microscopy settings.

The comparative evaluation of detection architectures provides additional insight into the suitability of transformer-based detectors for artifact-rich KOH microscopy. YOLOv11, a modern single-stage convolutional detector, achieved a mean recall of 0.842±0.012 but a lower mean precision of 0.812±0.016 (mean F1: 0.827±0.013; mean AP@0.50: 81.34±1.52%), suggesting a tendency to generate false positive detections in the presence of visually ambiguous artifacts. Faster R-CNN demonstrated the weakest overall performance, with a mean precision of 0.333±0.013 and a mean AP@0.50 of 51.54±1.23%, indicating that the region-proposal mechanism frequently nominated background structures, including keratin debris and synthetic fibres, as candidate fungal regions across all five test-set evaluations. Its inference speed of 255.4 ms per image also represents a substantial computational disadvantage compared to RT-DETR (41.9 ms). The comparatively high false positive rates observed for both CNN-based architectures may suggest that global contextual reasoning could be advantageous when discriminating morphologically similar structures in artifact-rich KOH microscopy. However, the present comparative evaluation assesses complete detection frameworks rather than isolating the contribution of individual architectural components. Therefore, the observed results should be interpreted as a comparative evaluation between detection architectures rather than as direct evidence for the superiority of the self-attention mechanism itself. These findings suggest that transformer-based end-to-end detection may offer practical advantages in artifact-rich microscopy settings, though validation on larger and more diverse datasets would be required to establish a definitive performance ranking across architectures.

Object-level localisation is also closely aligned with the way clinicians read KOH slides. In daily practice, fungal elements must be identified among numerous mimickers, including keratin debris, air bubbles, fibres and staining artifacts, many of which can resemble hyphae and contribute to diagnostic variability [4]. Less-experienced observers in particular may confuse hyphae with these structures, a well-documented limitation of KOH microscopy that contributes to diagnostic variability [5]. Moreover, routine KOH examination often requires manual scanning of multiple large microscopic fields, which is time-consuming and may further increase reader fatigue and variability [8]. By drawing bounding boxes around regions with a high probability of containing fungal elements while ignoring learned artifacts, the proposed model provides visual cues; whether these cues improve diagnostic decision-making requires prospective reader studies. Such visual localisation aligns with principles of explainable artificial intelligence, which emphasise transparency and human interpretability as key requirements for clinical deployment of AI systems [34]. In addition, the number and distribution of detected regions could in the future be used to approximate fungal burden or to monitor changes during treatment.

Quantitative analysis of the misclassified instances revealed that most false detections were associated with structures that closely resemble fungal hyphae, including synthetic fibres, scratch marks, overlapping cell borders, and sharp keratin edges. In addition, a small number of very faint or out-of-focus hyphal fragments with extremely low contrast against dense keratin backgrounds were occasionally missed. These cases represent the technical edge conditions encountered during the detection of subtle biological structures in heterogeneous KOH preparations. Overall, the quantitative results support the technical feasibility of the proposed approach. The low standard deviation of AP@0.50:0.95 across the five test-set evaluations (76.85±1.22%) confirms that spatial localisation quality is stable across the five independently trained models and that the results are not driven by a particularly favourable model initialisation. The near-zero false-negative rate on the independent test set (0.2±0.4 missed positive cases) is an encouraging preliminary finding for screening-oriented applications; however, it should be interpreted within the constraints of the single-centre retrospective design, and prospective validation is required before any inference about clinical reliability can be drawn. Evaluating AP across multiple IoU thresholds provides a more stringent measure of spatial agreement than a single-threshold metric and is consistent with current practice in object detection. The performance levels observed here are comparable to those reported for other microscopic detection tasks, including leukocyte detection, parasitic egg localisation, and hyphae detection in KOH images [25,29,30,31].

**Figure 4:**
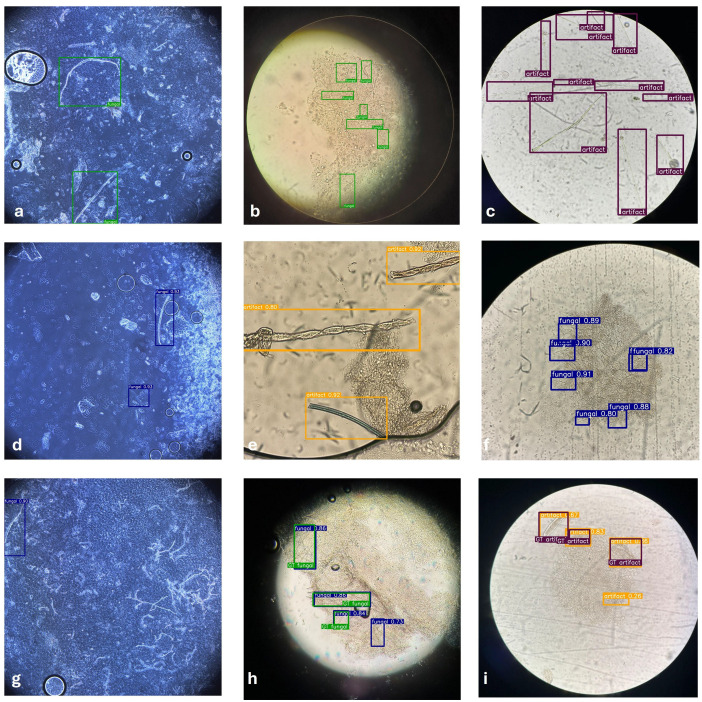
Qualitative examples of model predictions in KOH microscopy images. (**a**–**c**) Ground truth annotations showing fungal elements (green bounding boxes) and artifacts (purple bounding boxes). (**d**–**f**) Model predictions with confidence scores, including examples of correct discrimination between fungal structures (blue bounding boxes) and artifact mimics (yellow bounding boxes). (**g**–**i**) Overlay of ground truth and model predictions illustrating correct detections and representative misclassifications.

To qualitatively evaluate the model’s performance, a comparative failure analysis was conducted across different detection scenarios in Figure 4. The top row, comprising Figure 4a–c, illustrates the expert-verified ground truth (GT) annotations, highlighting the high density of fungal structures. The middle row shows the model’s independent inference; while Figure 4d,f demonstrate high-confidence detections, Figure 4e identifies “mimickers”, non-fungal artifacts that visually resemble hyphae, and labels them as artifacts to prevent diagnostic confusion.

The bottom row (Figure 4g–i) specifically illustrates instances of misclassification and the limitations of the detection framework. In Figure 4g, the model exhibits a false positive error by identifying a non-fungal structure as a hypha, which would lead to a healthy sample being incorrectly flagged as infected (dermatophytosis). Similarly, Figure 4h reveals a critical misclassification where the model identifies an ovoid, non-fungal structure as dermatophytosis, further demonstrating the system’s tendency to over-predict in the presence of ambiguous biological morphology. Finally, Figure 4i confirms that certain synthetic fibres and sharp keratin edges continue to trigger false detections despite the multi-class training strategy. These instances represent technical bottlenecks where the high morphological similarity between debris and true hyphae limits the model’s precision in complex KOH preparations.

Several characteristics of the dataset and training strategy have likely contributed to these results. The images were acquired under routine conditions and deliberately included a wide range of KOH dissolution stages, from partially cleared preparations with abundant keratin to more transparent fields. This heterogeneity introduces considerable noise and artifacts, which makes the task more challenging for the model but also increases the dataset’s relevance to real-world practice, where slides are rarely ideal. The use of high-resolution inputs and a transformer-based backbone allowed the network to combine local shape cues with broader contextual information from the surrounding tissue. This combination of fine-grained feature extraction and contextual modelling has also been shown to improve the detection of small, low-contrast structures in other microscopic imaging tasks [6].

This study has several limitations. The dataset originates from a single centre, and its moderate size, while mitigated by COCO-based pretraining and five-fold cross-validation, nonetheless restricts generalisability; external multi-centre validation is required to confirm performance across diverse clinical settings. Regarding class definitions, while the current model employs a multi-class strategy to distinguish fungal elements from artifacts, it treats all fungal species as a single category. Future extensions should therefore incorporate richer annotation schemes to differentiate specific fungal morphologies. A critical clinical consideration is the differential diagnosis of dermatophytosis. As emphasized by recent studies, nail unit malignancies-specifically subungual melanoma and squamous cell carcinoma-frequently mimic the clinical presentation of dermatophytosis through features like hyperkeratosis and dystrophic discoloration [35,36]. Since the proposed model is trained to detect fungal presence but not to exclude these mimickers, relying solely on automated screening could obscure concurrent malignancies. Therefore, future work aims to integrate anomaly detection mechanisms to flag atypical patterns for expert review. Finally, prospective reader studies will be essential to translate these image-level performance gains into tangible improvements in clinical diagnostic accuracy and reporting efficiency.

In conclusion, this study demonstrates that a transformer-based detection framework, reinforced by a multi-class training strategy, can reliably identify fungal elements even within the complex, artifact-laden environment of routine KOH microscopy. By translating neural network predictions into interpretable bounding-box overlays, the system provides localised visual cues, which may support human-in-the-loop review in future clinical workflows. Ultimately, this localisation capability may support the future integration of AI-based decision-support systems into clinical microscopy workflows, pending prospective validation, thereby potentially contributing to more consistent diagnostic assessment in dermatomycology.

## Figures and Tables

**Table 1:** Descriptive statistics of the microscopy dataset and annotation categories used for model training and evaluation.

Category	Component	Count / Value
Images	Total Microscopic Frames	2540
Resolution	Pixel Dimensions	2048×2048
Annotations	Fungal Elements (Positive Class)	631
	Confounding Artifacts (Negative Class)	381

**Table 2:** Object-level detection and image-level classification performance. All metrics: mean ± SD across five fold-level models evaluated on the independent held-out test set (n=254; 89 positive, 165 negative). A case was positive when ≥1 fungal element exceeded the 0.25 confidence threshold.

Level	Metric	RT-DETR	YOLOv11	Faster R-CNN
Object-Level	Precision	0.921±0.014	0.812±0.016	0.333±0.013
	Recall	0.831±0.011	0.842±0.012	0.800±0.008
	F1-score	0.874±0.012	0.827±0.013	0.470±0.015
	AP@0.50 (%)	89.73±1.48	81.34±1.52	51.54±1.23
	AP@0.50:0.95 (%)	76.85±1.22	61.28±1.41	27.47±1.01
	Mean IoU	0.838±0.009	0.846±0.008	0.750±0.008
Image-Level	Accuracy	0.976±0.008	0.886±0.012	0.765±0.018
	Sensitivity	0.989±0.022	0.767±0.014	0.522±0.021
	Specificity	0.970±0.005	0.951±0.008	0.896±0.013
	F1-score	0.967±0.012	0.826±0.013	0.610±0.019
	Missed Cases (FN)	0.2±0.4	4.2±1.1	8.6±1.5

SD: standard deviation across five fold-level models evaluated on the independent test set. AP: average precision. IoU: intersection over union. FN: mean false negatives (missed positive cases).

## Data Availability

The datasets generated and analyzed during the current study are available from the corresponding author upon reasonable request. Raw microscopic images are not publicly available due to ethical and privacy considerations associated with human-derived biological material.
